# Supplementary material for: Causal Relationship between Aging and Anorexia Nervosa: A White-Matter-Microstructure-Mediated Mendelian Randomization Analysis
Source: Biomedicines. 2024 Aug 16;12(8):1874. doi: 10.3390/biomedicines12081874 (PMC11351342; doi:10.3390/biomedicines12081874)
Supplement: Supplementary file 1 [file biomedicines-12-01874-s001.zip › Supplementary Figure/Figure S4/Singe SNP plot of MR analysis for TL to ubm-b-1867.pdf]

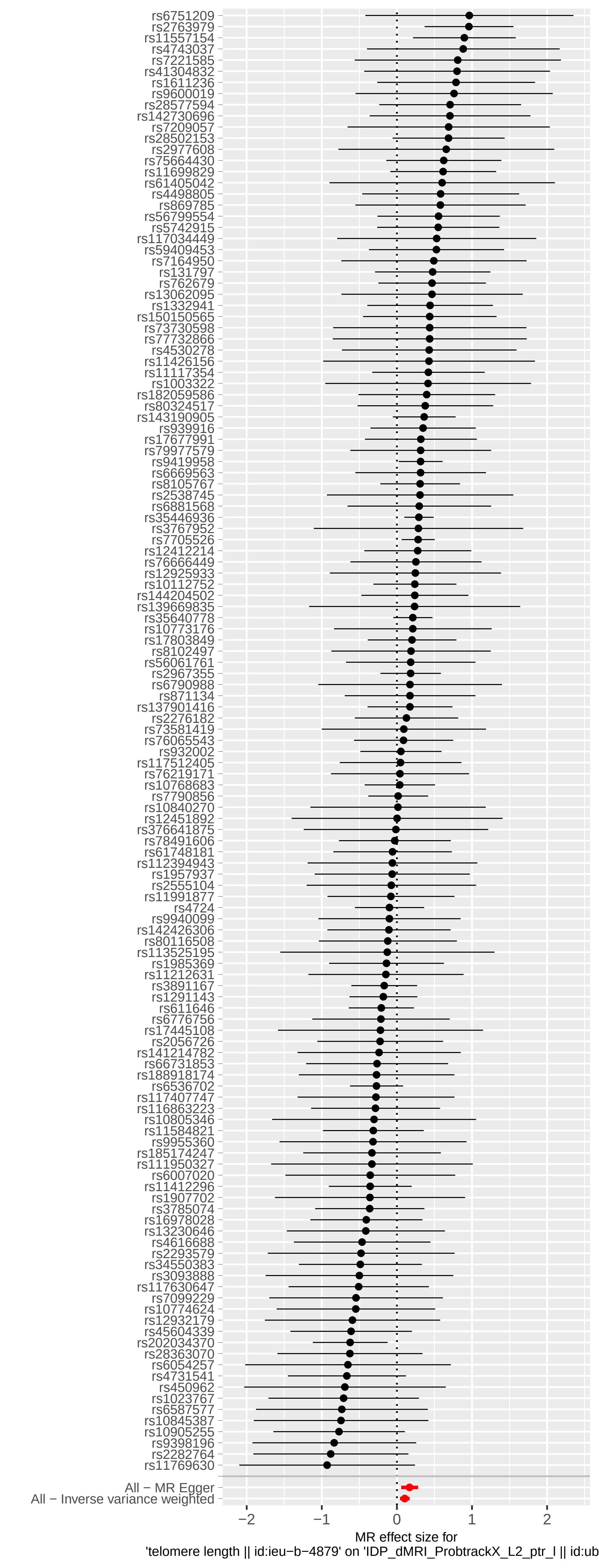

rs6751209
rs2763979
rs11557154
rs4743037
rs7221585
rs41304832
rs1611236
rs9600019
rs28577594
rs142730696
rs7209057
rs28502153
rs2977608
rs75664430
rs11699829
rs61405042
rs4498805
rs869785
rs56799554
rs5742915
rs117034449
rs59409453
rs7164950
rs131797
rs762679
rs13062095
rs1332941
rs150150565
rs73730598
rs77732866
rs4530278
rs11426156
rs11117354
rs1003322
rs182059586
rs80324517
rs143190905
rs939916
rs17677991
rs79977579
rs9419958
rs6669563
rs8105767
rs2538745
rs6881568
rs35446936
rs3767952
rs7705526
rs12412214
rs76666449
rs12925933
rs10112752
rs144204502
rs139669835
rs35640778
rs10773176
rs17803849
rs8102497
rs56061761
rs2967355
rs6790988
rs871134
rs137901416
rs2276182
rs73581419
rs76065543
rs932002
rs117512405
rs76219171
rs10768683
rs7790856
rs10840270
rs12451892
rs376641875
rs78491606
rs61748181
rs112394943
rs1957937
rs2555104
rs11991877
rs4724
rs9940099
rs142426306
rs80116508
rs113525195
rs1985369
rs11212631
rs3891167
rs1291143
rs611646
rs6776756
rs17445108
rs2056726
rs141214782
rs66731853
rs188918174
rs6536702
rs117407747
rs116863223
rs10805346
rs11584821
rs9955360
rs185174247
rs111950327
rs6007020
rs11412296
rs1907702
rs3785074
rs16978028
rs13230646
rs4616688
rs2293579
rs34550383
rs3093888
rs117630647
rs7099229
rs10774624
rs12932179
rs45604339
rs202034370
rs28363070
rs6054257
rs4731541
rs450962
rs1023767
rs6587577
rs10845387
rs10905255
rs9398196
rs2282764
rs11769630
All − MR Egger
All − Inverse variance weighted
−2
−1
0
1
2
MR effect size for
'telomere length || id:ieu−b−4879' on 'IDP_dMRI_ProbtrackX_L2_ptr_l || id:ub
